# Effects of Vitamin C Supplementation on Total Antioxidant Status, Inflammation, and Histopathological Changes in Aged Rats

**DOI:** 10.1002/jbt.70324

**Published:** 2025-05-30

**Authors:** Mehmet Başeğmez, Abdullah Eryavuz, Hasan Hüseyin Demirel

**Affiliations:** ^1^ Department of Veterinary, Laboratory and Veterinary Health Program, Acıpayam Vocational High School Pamukkale University Denizli Turkey; ^2^ Department of Physiology, Faculty of Veterinary Medicine Afyon Kocatepe University Afyonkarahisar Turkey; ^3^ Department of Veterinary, Laboratory and Veterinary Health Program, Bayat Vocational High School Afyon Kocatepe University Afyonkarahisar Turkey

**Keywords:** gender, pro‐inflammatory cytokines, rat, total antioxidant, vitamin C

## Abstract

This study aims to determine the effect of orally administered vitamin C (Vit C) supplementation on physiological and histopathological changes in aged rats of different genders. A total of 28 Sprague‐Dawley aged male and female rats were randomly divided into four groups of seven animals per group. The study groups included the aged male control (MC), aged male with Vit C (MVC) (500 mg/kg vitamin C, orally) supplementation, female aged control (FC), and female aged with vitamin C (FVC) (500 mg/kg vitamin C, orally) supplementation groups. At the end of the study, which lasted 31 days, blood, brain, heart, liver, and kidney tissues were collected from rats under ketamine (87 mg/kg) and xylazine (13 mg/kg) anesthesia. The results indicated that although Vit C supplementation had no effect on serum Vit C levels, gender had an effect on serum Vit C levels (*p* < 0.05). However, Vit C supplementation and gender did not affect serum IL‐6, IL‐1β, TOS, and OSI levels (*p* > 0.05). Vit C supplementation, without the effect of gender, significantly increased TNF‐α levels in MVC groups compared to MC groups (*p* < 0.05), while it significantly decreased them in FVC groups compared to FC groups (*p* < 0.05). In addition, Vit C significantly reduced histopathological alterations in brain, heart, and liver tissues associated with aging, including oxidative stress and inflammation. In conclusion, it was observed that orally administered 500 mg/kg Vit C supplementation to old rats is not an effective way to increase the Vit C pool in the body, but gender has an impact on the blood Vit C concentrations.

## Graphical abstract

In this study, 500 mg/kg vitamin C was administered orally to aged rats of both genders. The effect of supplementation on serum vitamin C levels was not detected, but the effect of gender was. Gender and vitamin C supplementation had no effect on serum IL‐6 and IL‐1β levels; however, gender did affect TNF‐α and TAS levels.

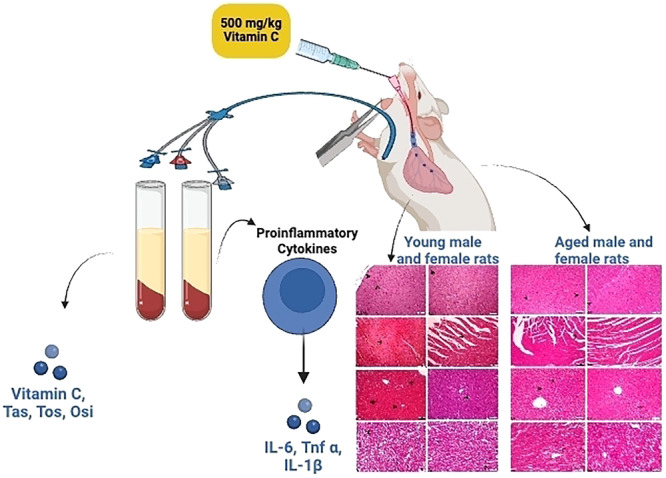

## 1 Introduction

Aging is a complex process that affects various physiological functions. It is accepted that the aging process weakens the immune response and creates a greater susceptibility to infections [1]. The fact that the aging process is re‐associated with increased oxidative damage has led to the hypothesis that reducing oxidative stress through antioxidant dietary factors can prolong life expectancy [2].

Vitamins and minerals are micronutrients in the organic structure that do not provide energy directly but are essential for the maintenance of metabolic functions, growth, and general health by contributing to the vital reactions of the organism. Aging may cause more requirement of some vitamins and minerals in people and animals [3]. In recent years, the individual use of vitamin and mineral supplements has increased, especially to protect against diseases and alleviate their effects, including elderly peoples [3, 4]. While vitamin C plays an antioxidant role in various biochemical and physiological processes of the body, it has also been reported to be effective in preventing diseases [5, 6]. It is stated that immunity is weakened in chronic deficiency [7]. These reports suggested that vitamin C allows a healthier life depending on its amount in many biological functions [8, 9, 10]. In healthy people, consumption of 200–250 mg of vitamin C twice a day has been shown to provide the highest dose associated with full bioavailability [11]. It has been reported that vitamin C taken in excess or not absorbed from the gastrointestinal tract has no clear benefit; its plasma concentrations are close to saturation at 400 mg per day; oral doses of vitamin C above 500 mg have almost no effect on the body pool; and it is completely excreted in the urine [11].

Dietary intake is the most critical variable that determines the sustainability of vitamin C in the body pool. The absorption, distribution, metabolism, and excretion of vitamin C are highly complex. The sodium‐dependent vitamin C transporters (SVCTs), SVCT1 and SVCT2, are responsible for the absorption of vitamin C in the intestine, its distribution to tissues, and its reabsorption by the kidneys [12, 13]. Physiological and metabolic factors may vary depending on others, such as disease, genetics, physical activity, malnutrition, and aging [3, 6]. In previous studies, it has been observed that any acute illness could result in a drop‐in plasma and cellular levels of vitamin C, possibly due to decreased intake and absorption, increased metabolism, and even redistribution [14, 15]. Although most mammals, including rats, synthesize vitamin C from glucose, humans and other primates have to meet their daily requirements through diets because they lack the enzyme L‐gulono‐g lactonoxidase, a rate‐limiting enzyme involved in vitamin C synthesis [5, 16]. Vitamin C is crucial in many stressful situations involving the immune system and inflammatory processes [17]. It controls the release of pro‐inflammatory cytokines such as interleukin 6 (IL‐6) and tumor necrosis factor (TNF‐α) [18]. Recent research suggests that vitamin C, an enzyme cofactor and antioxidant, can hasten the resolution of issues related to inflammation, oxidative stress, and microvascular dysfunction [19, 20].

The purpose of this study was to determine the physiological, biochemical, and histopathological effects of oral vitamin C supplementation on aged rats of different genders. We also investigated how orally administered vitamin C supplementation affects age‐related organ damage in aged rats of different genders.

## 2 Materials and Methods

### 2.1 Chemical

Vitamin C was purchased from Carlo Erba Reagent, France. All reagents and materials were acquired from commercial suppliers or prepared using published procedures.

### 2.2 Animals and Experimental Model

Twenty‐eight Sprague‐Dawley male and female aged rats (over 18 months and 450–500 mg in weight) were obtained from Anadolu University Experimental Animals Research and Application Unit, Eskisehir, Turkey. All rats underwent a quarantine period lasting 1 week (7 days). Rats were housed in polypropylene rat cages, in a 12‐h light–dark cycle, at an ambient temperature of 23 ± 1°C room temperature, and the application process lasted 30 days. The rats were provided with an ad libitum supply of food and water throughout the study. Test procedures were performed in accordance with the Guide for the Care and Use of Laboratory Animals. Pamukkale University's Animal Research ethics committee approved this study (protocol number PAUHDEK‐2019/37). The oral dose of vitamin C was determined in rats as 500 mg/body weight [11].

Rats were divided into four experimental groups, each containing seven rats (*n* = 7): aged male control (MC), aged male with vitamin C (MVC) (500 mg/kg vitamin C, orally), aged female control (FC), and aged female with vitamin C (FVC) (500 mg/kg vitamin C, orally). The experimental plan is given in Table 1.

**TABLE 1:** Groups, numbers of animals in groups, and application method.

Groups (n:7)	1st–30th day	31st day
Aged male control group (MC)	The rats were administered 1 mL of oral saline for 30 days.	On day 31 of the study, rats in all groups were anesthetized and euthanized.
Aged male vitamin C group (MVC)	The rats were given 500 mg/kg of oral vitamin C daily for 30 days.	
Aged female control group (FC)	The rats were administered 1 mL of oral saline for 30 days.	
Aged female vitamin C group (FVC)	The rats were given 500 mg/kg of oral vitamin C daily for 30 days.	

### 2.3 Collection of Blood and Tissue Samples

At the end of the study (Day 31), blood, brain, heart, liver, and kidney tissues were collected from rats under ketamine (87 mg/kg) and xylazine (13 mg/kg) anesthesia. TNF‐α, IL‐6, interleukin 1β (IL‐1β), total antioxidant status (TAS), total oxidant status (TOS), and vitamin C levels were measured in blood serum samples. Serum samples taken for biochemical analysis were stored in the freezer at −80°C, and tissue samples were fixed with 10% formalin for histopathological examination.

### 2.4 Measurement of Vitamin C, TNF‐α, IL‐6, IL‐1β

Serum vitamin C, TNF‐a, IL‐6, and IL‐1β levels were determined using commercial rat ELISA kits from Bioassay Technology Laboratory (Cat. No E0612Ra, Cat. No E0764Ra, Cat. No E0135Ra, and Cat. No BMS630 respectively), according to the manufacturer's instruction. The degree of enzymatic degradation of the substrate following the color change in the well was determined by absorbance measurements at 450 nm.

### 2.5 Measurement of TAS, TOS, and Oxidative Stress Index

The serum's TAS was determined using the Erel method [21] and a TAS kit (Rel Assay Diagnostics, Gaziantep, Turkey). Total antioxidant status value of the serum samples was expressed as mmol Trolox equivalent/L (mmol Trolox eq/L). The serum's TOS was determined using the Erel TOS method [22] and a TOS kit (Rel Assay Diagnostics, Gaziantep, Turkey). Total oxidant status value of the serum samples was expressed in μmoL H_2_O_2_ equivalent/L (μmol H2O2 eq/L).

The oxidative stress index (OSI) was defined as the TOS‐to‐TAS ratio and was calculated as follows: OSI (arbitrary unit) = (TOS, μmol H2O2 eq/L)/(TAS, μmol Trolox eq/L) [23].

### 2.6 Histopathological Analysis

Brain, heart, liver, and kidney tissues of aged rats of different genders were collected and fixed in 10% formaldehyde solution. Subsequently, these tissues were analyzed by histological follow‐up methods and embedded in paraffin blocks. Paraffin sections of 5–6 μm thickness were then prepared and stained with hematoxylin and eosin. They were then examined using a Nikon Eclipse Ci light microscope equipped with a Nikon DS FI3 microscopic digital camera system. Pathological analyses were independently evaluated by another pathologist through blind review. The histopathological changes found in the brain, heart, liver, and kidney of aged male and female rats from different groups were numbered as 0, 1, 2, and 3. Then statistical analyses were performed, and the results are presented in Table 2.

**TABLE 2:** Effect of vitamin C supplementation on histopathological alterations of aged male and female rats (*n* = 7, mean ± SE).

Histopathological changes		MC	MVC	FC	FVC
Brain	Hyperemia in the vessels	1.00 ± 0.03^a^	0.43 ± 0.01^b^	1.00 ± 0.03^a^	0.28 ± 0.01^b^
	Vacuolization in neurons	1.00 ± 0.03^a^	0.57 ± 0.20^b^	1.00 ± 0.03^a^	0.29 ± 0.01^b^
Heat	Hyaline degeneration in the myocardium	1.00 ± 0.02^a^	0.29 ± 0.00^b^	1.00 ± 0.03^a^	0.83 ± 0.01^a^
Liver	Vacuolar degeneration in hepatocytes	0.86 ± 0.01^a^	0.29 ± 0.00^b^	0.86 ± 0.01^a^	0.29 ± 0.01^b^
	Sinusoidal dilatation and hyperemia	1.00 ± 0.03^a^	0.43 ± 0.01^b^	1.00 ± 0.02^a^	0.29 ± 0.01^b^
Kidney	Dilatation of the glomerulus Bowman capsule	0.00 ± 0.00^b^	0.00 ± 0.00^b^	0.86 ± 0.01^a^	0.79 ± 0.01^a^

*Note:* 0, no observed changes; 1, mild changes; 2, moderate changes; 3, severe changes. The values were expressed as means ± SEM.

Abbreviations: FC; aged female control, FVC; aged female vitamin C, MC; aged male control, MVC; aged male vitamin C.

^a,b^In the same line values with different letters show statistically significant differences in brain, hearth, liver and kidney tissue (*p* < 0.05).

### 2.7 Statistical Analysis

Statistical analysis was carried out using SPSS 25.0 (SPSS Inc., Chicago, IL, USA). GraphPad Prism 9.5.0 (GraphPad Software, San Diego, CA, USA) was used for the graphical presentation. Results are provided as mean ± standard error of the mean (SEM). The suitability of the data for a normal distribution was examined with the Shapiro‐Wilk test, and the assumption of variance homogeneity was examined with the Levene statistic. The Duncan post hoc test and one‐way analysis of variance (ANOVA) were used to compare multiple groups. Statistical significance was determined at an acceptable level of *p* < 0.05.

## 3 Results

### 3.1 Biochemical Parameter Changes

**FIGURE 1:**
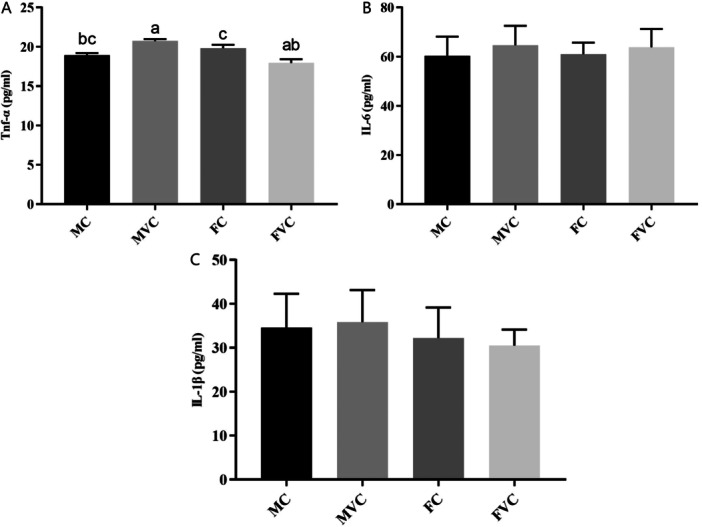
The effects of vitamin C supplementation on TNF‐α, IL‐6, and IL‐1β levels in the serum of aged male and female rats (*n* = 7 for each group). (A) TNF‐α levels in serum, (B) IL‐6 levels in serum, (C) IL‐1β levels in serum. ^a,b,c^Different letters indicate statistical differences between groups (*p* < 0.05). The values were expressed as means ± SEM. FC; aged female control, FVC; aged female vitamin C; MC; aged male control, MVC; aged male vitamin C.

**FIGURE 2:**
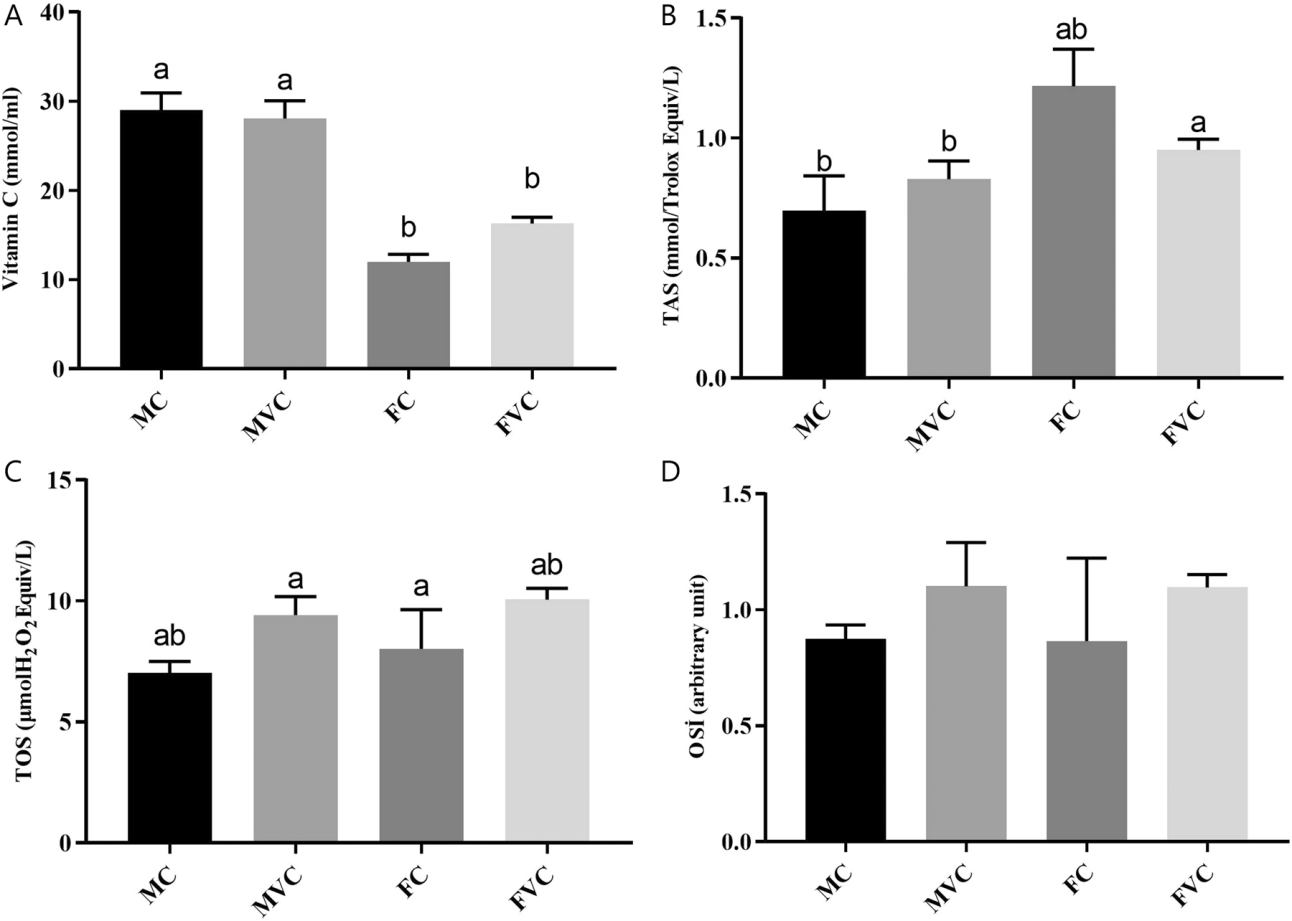
The effects of vitamin C supplementation on Vitamin C, TAS, TOS, and OSİ levels in the serum of aged male and female rats (*n* = 7 for each group). (A) vitamin C levels in serum, (B) TAS levels in serum, (C) TOS levels in serum, (D) OSİ levels. ^a,b^Different letters indicate statistical differences between groups (*p* < 0.05). The values were expressed as means ± SEM. FC; aged female control, FVC; aged female vitamin C, MC; aged male control, MVC; aged male vitamin C.

As shown in Figures 1 and 2, vitamin C supplementation has no effect on the serum IL‐6, IL‐1β, TAS, TOS, and OSI concentrations in aged rats (*p* > 0.05). However, as demonstrated in Figure 2, gender has an effect on serum vitamin C levels (*p* < 0.05). Serum vitamin C levels were significantly higher in the MC and MVC groups compared to the FC and FVC groups (*p* < 0.05).

### 3.2 Histopathological Results

**FIGURE 3:**
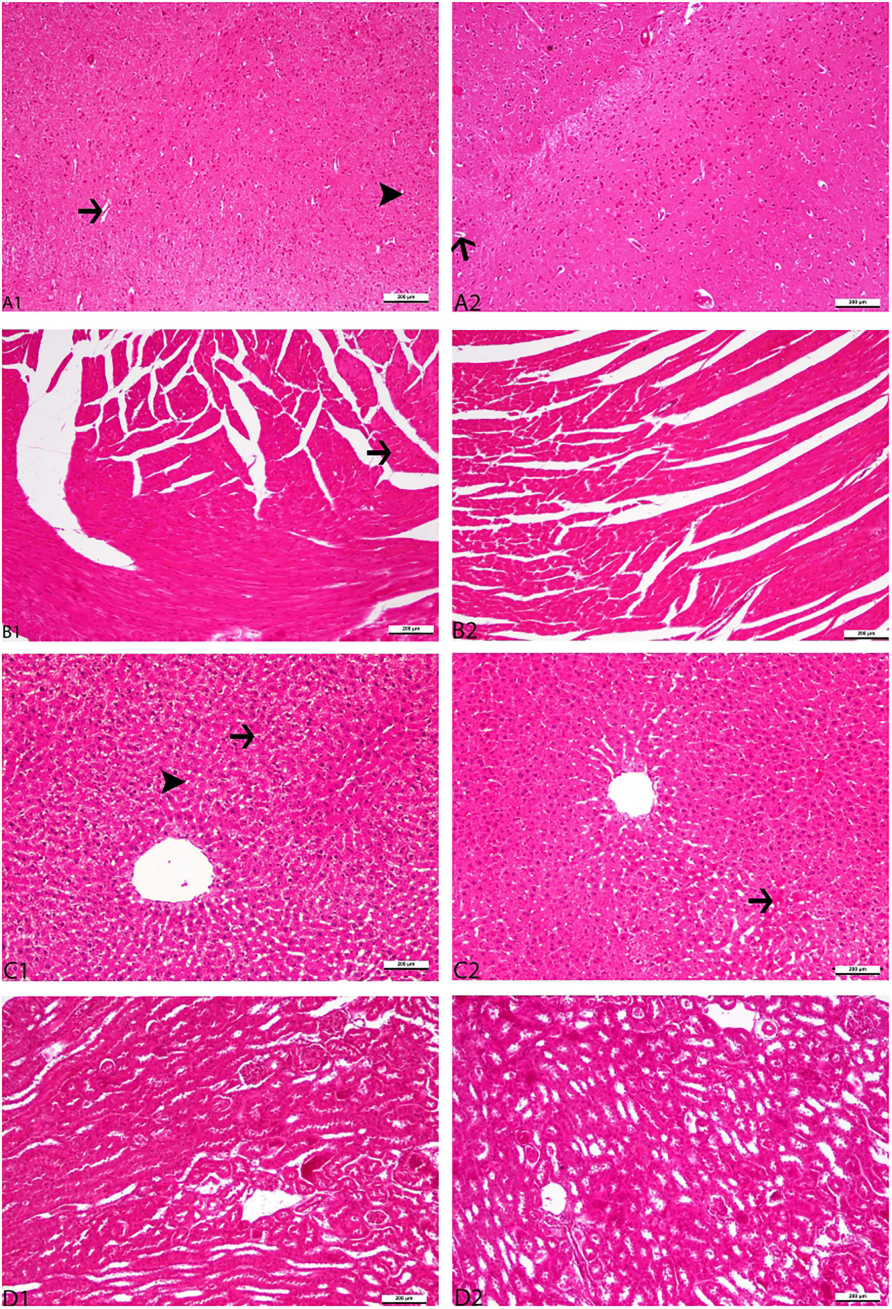
Histopathological appearance of vitamin C supplementation in the brain, heart, liver, and kidney tissues of aged male rats. Panels respectively: control (A1, B1, C1, D1), 500 mg/kg vitamin C (A2, B2, C2, D2). Representative Figure were stained with H&E. The original magnification was 20, and the scale bars represent 200 µm. Thin arrows indicate hyperemia in the vessels of the brain (Figure 3A1, A2). Arrowheads indicate vacuolization formation in neurons in the brain (Figure 3A1). The thin arrow indicates hyaline degeneration formation in the myocardium (Figure 3B1). The thin arrow shows sinusoidal dilatation and hyperemia in the liver (Figure 3C1, C2). The arrowhead indicates the formation of vacuolar degeneration in hepatocytes (Figure 3C1).

**FIGURE 4:**
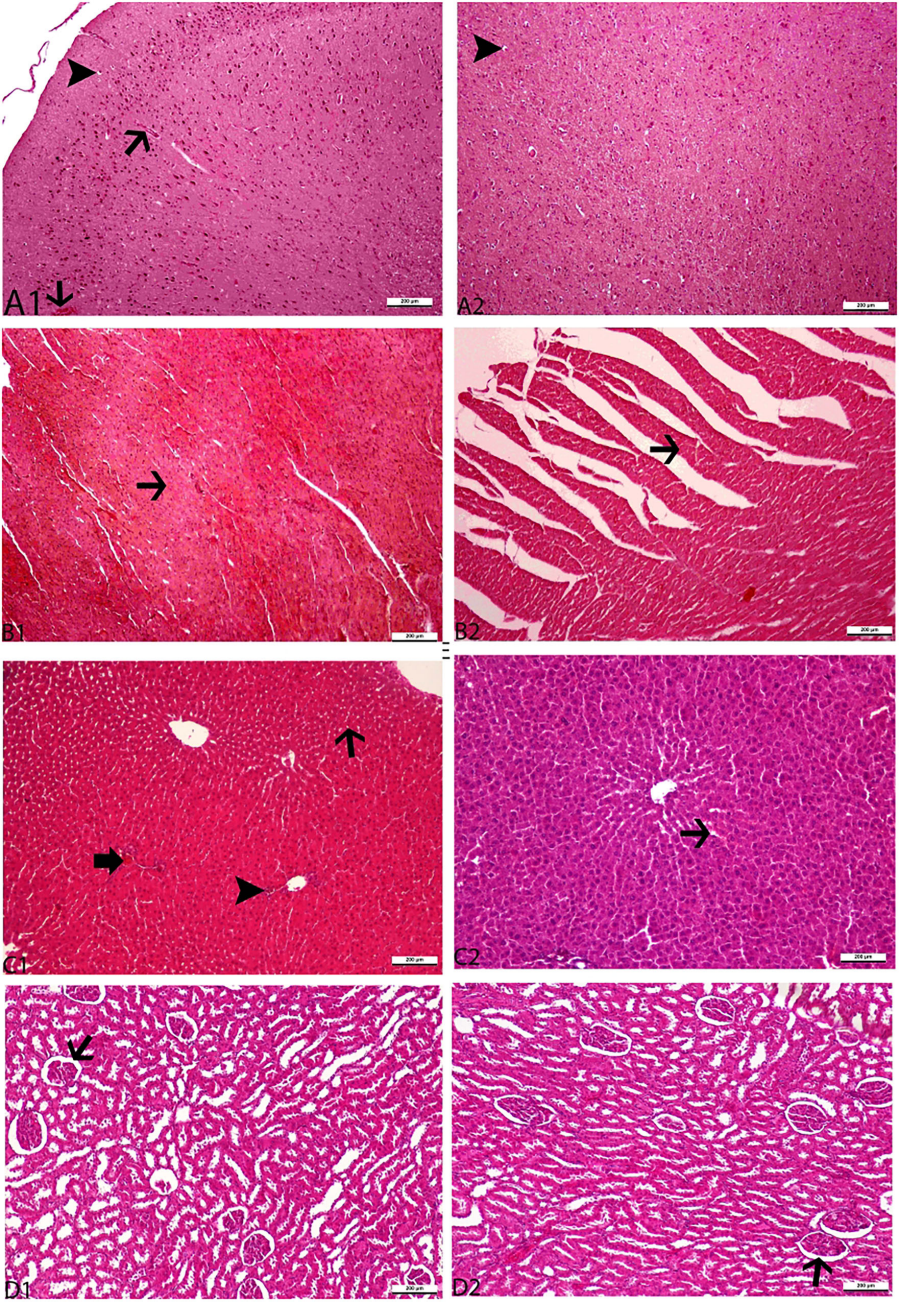
Histopathological appearance of vitamin C supplementation in the brain, heart, liver, and kidney tissues of aged female rats. Panels respectively: control (A1, B1, C1, D1), 500 mg/kg vitamin C (A2, B2, C2, D2). Representative figüre was stained with H&E. The original magnification was 20, and the scale bars represent 200 µm. Thin arrows indicate hyperemia in the vessels of the brain (Figure 4 A1). Arrowheads indicate vacuolization formation in neurons in the brain (Figure 4 A1, A2). The thin arrow indicates hyaline degeneration formation in the myocardium (Figure 4 B1, B2). The thin arrow shows sinusoidal dilatation and hyperemia in the liver (Figure 4 C1, C2). The arrowhead indicates the formation of MNH infiltration in hepatocytes (Figure 4 C1). The thick arrow indicates the formation in the liver of vacuolar degeneration in hepatocytes (Figure 4 C1). The thin arrow indicates enlargement of Glomerulus Bowman's capsule (Figure 4 D1, D2).

Histopathological changes in the brain, heart, liver, and kidney of aged male and female rats in the experimental groups were described in detail and shown in Figures 3 and 4, respectively. As shown in Figures 3A1 and 4A1, vacuolization in neurons and hyperemia in brain vessels were observed in the MC and FC groups. Vitamin C supplementation significantly reduced hyperemia in brain vessels (Figures 3A2 and 4A2) and vacuolization in neurons (Figures 3A2 and 4A2) in aged rats in the MVC and FVC groups. The heart tissue in the MC and FC groups showed the development of hyaline degeneration, as shown in Figures 3B1 and 4B1. Vitamin C reduced cardiac tissue organ damage in the MVC group (Figure 3B2) but had no effect in the FVC group (Figure 4B2).

As shown in Figures 3C1 and 4C1, hepatocytes in the MC and FC groups demonstrated vacuolar degeneration, sinusoidal dilatation, and hyperemia. Vitamin C decreased organ damage in the liver tissue of aged rats in the MVC and FVC groups, as shown in Figures 3C2 and 4C2. As shown in Figure 4D1, the kidney Bowman capsule space was enlarged in the FC group. However, as seen in Figure 4D2, the FVC group seemed to have less damage to kidney tissue, but the difference was not statistically significant.

## 4 Discussion

Reactive oxygen species (ROS) play key roles in many physiologic and pathogenic processes. Excess ROS generation has damaged various cell components and triggered the activation of specific signaling pathways. These effects can influence numerous cellular processes linked to aging and the development of age‐related disease [24]. Vitamin C could decrease oxidative stress during aging since it is an important nutritional antioxidant that protects tissues against the deleterious effects of ROS. In our study, aged male and female rats were given oral vitamin C (500 mg/kg) for 30 days, and the histopathological changes in the brain, heart, liver, and kidney were examined. TNF‐α, IL‐6, IL‐1β, Vit C, TAS, and TOS levels in the serum were also measured.

In this present study, giving rats 500 mg/kg of vitamin C did not change the serum vitamin C levels in aged rats of the same age and gender. Similarly, Levine et al. [11] reported that healthy volunteers excreted more than 500 mg/kg of vitamin C in urine without changing plasma levels. This suggests that when daily vitamin C intake through diet is sufficient, high‐dose oral supplementation does not significantly increase vitamin C levels in the blood or tissues. Also, the regulation of intestinal absorption and renal function of vitamin C in the body varies depending on the oral dosage. Previous studies have demonstrated that high‐dose oral vitamin C intake adversely affects the SVCT1 and SVCT2 transport mechanisms and leads to a reduction in vitamin C absorption efficiency [11, 25]. Indeed, this shows that vitamin C has tight control over both gastrointestinal absorption and transport systems, which influence vitamin C levels in tissues [8]. The result in this study showed that aged rats do not need to take high amounts of vitamin C supplements orally to maintain a healthy aging process.

The body's defense of vitamin C during low intakes and the limitation of blood levels during high intakes clearly demonstrate vitamin C homeostasis in the body [26, 27]. Kallner et al. [28] reported an inverse relationship between plasma vitamin C concentration and vitamin C half‐life level. Similarly, Hornig et al. [29] and MacDonald et al. [25] suggested that high doses of vitamin C intake may significantly reduce the percentage of vitamin absorption. Similarly, this study supports the report that oral vitamin C supplementation at pharmacologic doses did not cause an increase in blood vitamin C levels [30]. In the present study, the lack of effect of oral vitamin C supplementation on serum vitamin C levels may be explained by the achievement of homeostatic saturation in healthy aged rats, which is likely due to already high basal serum vitamin C levels. However, the study's lack of effect of oral vitamin C supplementation on serum vitamin C levels in the same age and gender groups raises questions about the physiological significance of this intervention. To understand the physiological significance of serum vitamin C levels in aged rats, further research investigating the contribution of vitamin C supplementation in various routes of administration, such as the parenteral route, to the aging process is needed

In the present study, aged female rats had lower serum vitamin C levels compared to aged male rats, regardless of the effect of supplementation. This result was not consistent with a previous report of no effect of gender on serum vitamin C levels in humans [31]. This was also inconsistent with the report that serum vitamin C levels were higher in women than men and that women achieved higher serum and plasma concentrations at equal vitamin C intake than men [32]. The reason for the difference between the results of this study and other studies may be that the rats used in the study, unlike humans, have the ability to synthesize vitamin C in the liver [33] and meet their daily normal vitamin C requirements in this way. The present study found that giving aged rats of both genders a vitamin C supplement did not alter the amount of vitamin C in their blood. This means that both aged male and aged female rats responded identically to oral vitamin C supplementation, and it did not help raise the low serum vitamin C levels of female rats.

Daily vitamin C requirements can vary significantly due to infections, increased inflammation, and metabolic diseases [19]; this may be especially true for malnutrition. Studies on animal models suggest that a loss of vitamin C may cause the neuroendocrine‐immune balance to get worse [34]. This may also be a reason why hormonal imbalances, mental disorders, and behavioral problems become more common with age [35]. In this present study, regardless of the effect of supplementation, older females appeared to have lower serum vitamin C levels than older males. This could be due to differences in vitamin C absorption and tissue intake in men and women, as well as different amounts of vitamin C receptors in the intestines and other tissues [36]. In addition, since hormones can affect vitamin C homeostasis in the body [36], gender hormones may have affected vitamin C absorption and pharmacokinetics differently in aged female rats than in aged male rats.

Vitamin C deficiency causes decreased immunity and increased susceptibility to infections [37]. This study found that vitamin C supplementation had no effect on IL‐6 and IL‐1β levels in the study groups. Similarly, Abdel‐Rahman et al. [38] demonstrate that vitamin C administration did not change the serum IL‐6 and IL‐1β levels. This may suggest that high oral doses of vitamin C supplementation make it very difficult to change the levels of IL‐6 and IL‐1β cytokines in the blood. Surprisingly, in the present study, vitamin C supplementation was found to increase TNF‐α levels in aged male rats while decreasing them in aged female rat groups. These results are consistent with the report that vitamin C supplementation decreases serum TNF‐α levels in aged female rats [39]. However, it is not consistent with the report that it increases serum TNF‐α levels in aged male rats [40]. Indeed, hormonal factors may affect vitamin C homeostasis [36, 41], so serum TNF‐α levels in aged female rats might be different from aged male rats.

In the present study, vitamin C supplementation showed that there was no statistical difference in TAS, TOS, and OSI parameters in rats of the same gender group. These results may be attributed to the absence of any significant effect of oral vitamin C supplementation on serum vitamin C levels. Oxidative stress occurs when homeostatic processes fail and free radical production exceeds the body's defensive capacity. Therefore, oxidative stress can be defined as an imbalance between the body's pro‐oxidants and antioxidants [42]. Vitamin C has an antioxidant effect at normal blood plasma levels but a pro‐oxidant effect at high concentrations [43] This means that the vitamin C supplements used in the present study did not reach serum levels that would cause antioxidant or pro‐oxidant effects.

In the present study, histopathological analysis showed that both the MC and FC groups had organ damage. Neurons had hyperemia and vacuolization, myocardium had hyaline degeneration, hepatocytes had vacuolar degeneration, and brain vessels had sinusoidal dilation and hyperemia. Badgujar et al. [44] found that vitamin C administration reduced histological damage in the brain tissue of mice. Similarly, in our current investigation, vitamin C supplementation notably decreased histopathological damage in the brain tissue of both elderly male and female rats. Furthermore, the study's results support the finding that vitamin C supplementation effectively decreased histopathological damage in the brain tissue of elderly rats [45]. In this study, vitamin C supplementation improved the pathological architecture of the liver in aged rats. This result was in line with the earlier study Ayman et al. [46] that vitamin C treatment improved the pathological architecture of the liver in rats. This study also supports the idea that vitamin C supplements may reduce damage to cardiac tissue due to a doxorubicin‐induced increase in proapoptotic proteins [47].

## 5 Conclusion

This study found that the levels of serum vitamin C, TNF‐α, and TAS levels were uniquely different in males and females. Supplementation with vitamin C had no effect on the serum levels identified for vitamin C, IL‐6, and IL‐1β irrespective of gender. We found that vitamin C supplementation reduced age‐related brain, heart, and liver organ damage in the MVC and FVC groups.

## Author Contributions

**Mehmet Başeğmez:** investigation, methodology, writing – original draft, writing – review and editing, data curation, resources, funding acquisition, conceptualization, visualization. **Abdullah Eryavuz:** project administration, writing – original draft, investigation, writing – review and editing, funding acquisition, conceptualization, validation. **Hasan Hüseyin Demirel:** investigation, methodology, visualization.

## Consent

All authors voluntarily participated in this research study. All authors have consent for the publication of the manuscript.

## Conflicts of Interest

The authors declare no conflicts of interest.

## Data Availability

The data that support the findings of this study are available on request from the corresponding author. The data are not publicly available due to privacy or ethical restrictions.
